# Supplementary material for: The organellar genomes of Silvetia siliquosa (Fucales, Phaeophyceae) and comparative analyses of the brown algae
Source: PLoS One. 2022 Jun 16;17(6):e0269631. doi: 10.1371/journal.pone.0269631 (PMC9202911; doi:10.1371/journal.pone.0269631)
Supplement: S1 Table — (DOCX) [file pone.0269631.s001.docx]

**S1 Table. The species used in the phylogenetic tree and their Genbank number.**

| Species | Genbank number | Species | Genbank number |
| --- | --- | --- | --- |
| *Cladosiphon okamuranus* | NC_040224 | *Heterosigma akashiwo* | NC_016738 |
| *Colpomenia peregrina* | KM244739 | *Laminaria digitata* | NC_004024 |
| *Costaria costata* | NC_023506 | *Macrocystis integrifolia* | NC_042669 |
| *Desmarestia viridis* | AY500367 | *Saccharina japonica* | NC_013476 |
| *Dictyopteris divaricata* | NC_043845 | *Sargassum fusiforme* | NC_024655 |
| *Dictyota dichotoma* | NC_007685 | *Sargassum horneri* | KJ938300 |
| *Ectocarpus siliculosus* | NC_030223 | *Sargassum thunbergii* | KP280065 |
| *Endarachne binghamiae* | MG488291 | *Silvetia siliquosa* | MW485980 |
| *Fucus distichus* | KY678904 | *Turbinaria ornata* | NC_027413 |
| *Fucus vesiculosus* | AY494079 | *Undaria pinnatifida* | KF319031 |
