# Supplementary material for: The organellar genomes of Silvetia siliquosa (Fucales, Phaeophyceae) and comparative analyses of the brown algae
Source: PLoS One. 2022 Jun 16;17(6):e0269631. doi: 10.1371/journal.pone.0269631 (PMC9202911; doi:10.1371/journal.pone.0269631)
Supplement: S2 Table — (DOCX) [file pone.0269631.s002.docx]

**S2 Table. Fossil constraints used in the MCMCtree analyses in this study.**

| Fossil constraints | Constraint Node | Min age (Ma) | References |
| --- | --- | --- | --- |
| Dictyotales | Crown with stem group Dictyotales | 99.6 | Silberfeld et al. 2010 |
| Fucales | Fucaceae and Sargassaceae | 13.0 | Starko et al. 2019 |

References:

Silberfeld T, Leigh JW, Verbruggen H, Cruaud C, Reviers BD, Rousseau F (2010) A multi-locus time-calibrated phylogeny of the brown algae (Heterokonta, Ochrophyta, Phaeophyceae): Investigating the evolutionary nature of the “brown algal crown radiation”. *Mol Phylogenet Evol* 56(2): 659-674.

Starko S, Gomez MS, Darby H, Demes KW, Kawai H, Yotsukura N, Lindstrom SC, Keeling PJ, Graham SW, Martone PT (2019) [A comprehensive kelp phylogeny sheds light on the evolution of an ecosystem](https://www.sciencedirect.com/science/article/pii/S1055790319300892). *Mol Phylogenet Evol* 136: 138-150.
