# Supplementary material for: The organellar genomes of Silvetia siliquosa (Fucales, Phaeophyceae) and comparative analyses of the brown algae
Source: PLoS One. 2022 Jun 16;17(6):e0269631. doi: 10.1371/journal.pone.0269631 (PMC9202911; doi:10.1371/journal.pone.0269631)
Supplement: S3 Table — (DOCX) [file pone.0269631.s003.docx]

**S3 Table. Mitochondrial genomes substitution rates in Phaeophyceae.**

| Gene | dN/dS | dN | dS |
| --- | --- | --- | --- |
| *atp6* | 0.0178 | 0.0552 | 3.1001 |
| *atp8* | 0.0397 | 0.0680 | 1.7124 |
| *atp9* | 0.0051 | 0.0043 | 0.8452 |
| *cob* | 0.0091 | 0.0314 | 3.4524 |
| *cox1* | 0.0059 | 0.0169 | 2.8679 |
| *cox2* | 0.0115 | 0.0277 | 2.4058 |
| *cox3* | 0.0217 | 0.0712 | 3.2822 |
| *nad1* | 0.0095 | 0.0277 | 2.9183 |
| *nad2* | 0.0222 | 0.0735 | 3.3102 |
| *nad3* | 0.0103 | 0.0326 | 3.1657 |
| *nad4* | 0.0082 | 0.0248 | 3.024 |
| *nad4L* | 0.0053 | 0.0163 | 3.0797 |
| *nad5* | 0.0143 | 0.0462 | 3.2292 |
| *nad6* | 0.018 | 0.0496 | 2.7548 |
| *nad7* | 0.0095 | 0.0337 | 3.5517 |
| *nad9* | 0.0231 | 0.0512 | 2.2176 |
| *nad11* | 0.0143 | 0.0547 | 3.8236 |
| *rpl2* | 0.0359 | 0.1132 | 3.1538 |
| *rpl5* | 0.0501 | 0.0989 | 1.9742 |
| *rpl6* | 0.0359 | 0.1102 | 3.0683 |
| *rpl14* | 0.0424 | 0.1999 | 4.7153 |
| *rpl16* | 0.022 | 0.0593 | 2.6986 |
| *rpl31* | 0.0431 | 0.1057 | 2.4529 |
| *rps2* | 0.056 | 0.2017 | 3.6016 |
| *rps3* | 0.0283 | 0.0897 | 3.1695 |
| *rps4* | 0.0191 | 0.0498 | 2.6074 |
| *rps7* | 0.0613 | 0.1911 | 3.1172 |
| *rps8* | 0.0461 | 0.1226 | 2.6586 |
| *rps10* | 0.0334 | 0.0714 | 2.1382 |
| *rps11* | 0.0557 | 0.2066 | 3.7089 |
| *rps12* | 0.0411 | 0.0960 | 2.3352 |
| *rps13* | 0.0663 | 0.1489 | 2.2457 |
| *rps14* | 0.0341 | 0.0865 | 2.5359 |
| *rps19* | 0.0508 | 0.0941 | 1.8533 |
| *tatC* | 0.0379 | 0.0904 | 2.3844 |

dN: nonsynonymous substitutions; dS: synonymous substitutions
