# Supplementary material for: The organellar genomes of Silvetia siliquosa (Fucales, Phaeophyceae) and comparative analyses of the brown algae
Source: PLoS One. 2022 Jun 16;17(6):e0269631. doi: 10.1371/journal.pone.0269631 (PMC9202911; doi:10.1371/journal.pone.0269631)
Supplement: S4 Table — (DOCX) [file pone.0269631.s004.docx]

**S4 Table. Chloroplast genomes substitution rates in Phaeophyceae.**

| Gene | dN/dS | dS | dN |  | Gene | dN/dS | dS | dN |
| --- | --- | --- | --- | --- | --- | --- | --- | --- |
| *ascF* | 0.027 | 2.2606 | 0.0610 |  | *psaL* | 0.0306 | 2.0656 | 0.0632 |
| *atpA* | 0.0149 | 2.2144 | 0.0330 |  | *psaM* | 0.0345 | 2.5258 | 0.0871 |
| *atpB* | 0.0116 | 2.0171 | 0.0234 |  | *psb28* | 0.0173 | 2.5034 | 0.0433 |
| *atpD* | 0.0389 | 2.1631 | 0.0841 |  | *psbA* | 0.0194 | 0.4345 | 0.0084 |
| *atpE* | 0.0415 | 1.7011 | 0.0706 |  | *psbB* | 0.0076 | 1.3362 | 0.0102 |
| *atpF* | 0.0504 | 2.0601 | 0.1038 |  | *psbC* | 0.0075 | 1.1119 | 0.0083 |
| *atpG* | 0.0385 | 2.0332 | 0.0783 |  | *psbD* | 0.0136 | 0.8958 | 0.0122 |
| *atpH* | 0.0198 | 0.9004 | 0.0178 |  | *psbE* | 0.0121 | 1.4336 | 0.0173 |
| *atpI* | 0.0275 | 1.8116 | 0.0498 |  | *psbF* | 0.0162 | 1.3868 | 0.0225 |
| *cbbx* | 0.0195 | 2.3022 | 0.0449 |  | *psbH* | 0.0082 | 1.6014 | 0.0131 |
| *ccs1* | 0.0301 | 2.6327 | 0.0792 |  | *psbI* | 0.012 | 2.6064 | 0.0313 |
| *ccsA* | 0.0429 | 2.0256 | 0.0869 |  | *psbJ* | 0.0095 | 0.7842 | 0.0074 |
| *chlB* | 0.0112 | 2.4478 | 0.0274 |  | *psbK* | 0.0465 | 1.3045 | 0.0607 |
| *chlI* | 0.0195 | 2.7036 | 0.0527 |  | *psbL* | 0.0071 | 1.0557 | 0.0075 |
| *chlL* | 0.0059 | 2.4478 | 0.0144 |  | *psbT* | 0.0255 | 1.1804 | 0.0301 |
| *chlN* | 0.023 | 2.3653 | 0.0544 |  | *psbV* | 0.0157 | 2.2966 | 0.0361 |
| *clpC* | 0.0091 | 2.2474 | 0.0205 |  | *psbX* | 0.1047 | 1.147 | 0.1201 |
| *dnaB* | 0.0399 | 2.3061 | 0.0920 |  | *psbY* | 0.0592 | 1.2633 | 0.0748 |
| *dnaK* | 0.0133 | 2.7669 | 0.0368 |  | *rbcL* | 0.0235 | 1.1895 | 0.0280 |
| *ftrB* | 0.047 | 2.1274 | 0.1000 |  | *rbcS* | 0.0479 | 1.5046 | 0.0721 |
| *ftsH* | 0.0117 | 2.3286 | 0.0272 |  | *rpl1* | 0.0426 | 1.6376 | 0.0698 |
| *groEL* | 0.0127 | 2.7579 | 0.0350 |  | *rpl2* | 0.0277 | 2.6293 | 0.0728 |
| *ilvB* | 0.0278 | 2.3401 | 0.0651 |  | *rpl3* | 0.0512 | 2.502 | 0.1281 |
| *ilvH* | 0.0387 | 2.338 | 0.0905 |  | *rpl4* | 0.0593 | 2.0264 | 0.1202 |
| *petA* | 0.0325 | 2.3348 | 0.0759 |  | *rpl5* | 0.0336 | 2.1198 | 0.0712 |
| *petB* | 0.0019 | 2.0612 | 0.0039 |  | *rpl6* | 0.0451 | 2.3094 | 0.1042 |
| *petD* | 0.0048 | 1.9197 | 0.0092 |  | *rpl9* | 0.0778 | 1.7463 | 0.1359 |
| *petF* | 0.0712 | 2.6064 | 0.1856 |  | *rpl11* | 0.0214 | 2.5915 | 0.0555 |
| *petG* | 0.0329 | 1.3109 | 0.0431 |  | *rpl12* | 0.043 | 2.1724 | 0.0934 |
| *petJ* | 0.0393 | 3.1509 | 0.1238 |  | *rpl13* | 0.0379 | 2.5464 | 0.0965 |
| *petM* | 0.0425 | 0.7268 | 0.0309 |  | *rpl14* | 0.008 | 2.2006 | 0.0176 |
| *petN* | 0.004 | 2.1353 | 0.0085 |  | *rpl16* | 0.0188 | 2.5929 | 0.0487 |
| *psaA* | 0.0098 | 1.6605 | 0.0163 |  | *rpl18* | 0.0333 | 2.6636 | 0.0887 |
| *psaB* | 0.0105 | 1.4401 | 0.0151 |  | *rpl19* | 0.0291 | 2.3874 | 0.0695 |
| *psaC* | 0.0038 | 1.652 | 0.0063 |  | *rpl20* | 0.0376 | 2.0743 | 0.0780 |
| *psaD* | 0.0144 | 2.1078 | 0.0303 |  | *rpl21* | 0.019 | 1.4064 | 0.0267 |
| *psaE* | 0.056 | 2.3071 | 0.1292 |  | *rpl22* | 0.0312 | 1.8087 | 0.0564 |
| *psaF* | 0.0478 | 1.7562 | 0.0839 |  | *rpl23* | 0.0492 | 1.6549 | 0.0814 |
| *psaI* | 0.0379 | 1.5608 | 0.0592 |  | *rpl24* | 0.0327 | 1.8236 | 0.0596 |
| *psaJ* | 0.0116 | 1.845 | 0.0214 |  | *rpl27* | 0.0331 | 2.0552 | 0.0680 |
| *rpl29* | 0.0333 | 1.2571 | 0.0419 |  | *rps19* | 0.024 | 1.5388 | 0.0369 |
| *rpl31* | 0.0273 | 2.1555 | 0.0588 |  | *rps20* | 0.0482 | 2.2979 | 0.1108 |
| *rpl33* | 0.0165 | 2.292 | 0.0378 |  | *secY* | 0.0273 | 2.4154 | 0.0659 |
| *rpl34* | 0.0574 | 1.6255 | 0.0933 |  | *sufC* | 0.0235 | 2.3613 | 0.0555 |
| *rpl35* | 0.0582 | 1.8214 | 0.1060 |  | *tatC* | 0.0192 | 1.8963 | 0.0364 |
| *rpl36* | 0.0484 | 1.2364 | 0.0598 |  | *thiG* | 0.0253 | 3.1399 | 0.0794 |
| *rpoA* | 0.0525 | 2.1844 | 0.1147 |  | *thiS* | 0.0809 | 2.1379 | 0.1730 |
| *rpoB* | 0.0374 | 2.2901 | 0.0856 |  | *tsf* | 0.0293 | 3.0118 | 0.0882 |
| *rpoC1* | 0.0202 | 2.4539 | 0.0496 |  | *tufA* | 0.0193 | 2.0131 | 0.0389 |
| *rpoC2* | 0.0138 | 1.5903 | 0.0219 |  | *ycf3* | 0.0067 | 2.7063 | 0.0181 |
| *rps1* | 0.0471 | 2.9304 | 0.1380 |  | *ycf4* | 0.0296 | 2.2732 | 0.0673 |
| *rps2* | 0.0146 | 1.7271 | 0.0252 |  | *ycf12* | 0.0261 | 1.0459 | 0.0273 |
| *rps3* | 0.0368 | 2.3375 | 0.0860 |  | *ycf19* | 0.0237 | 2.2377 | 0.0530 |
| *rps4* | 0.0372 | 1.8958 | 0.0705 |  | *ycf33* | 0.0908 | 2.0996 | 0.1906 |
| *rps5* | 0.0202 | 2.2619 | 0.0457 |  | *ycf34* | 0.0411 | 2.1438 | 0.0881 |
| *rps7* | 0.0154 | 3.0437 | 0.0469 |  | *ycf35* | 0.0335 | 1.6787 | 0.0562 |
| *rps8* | 0.0159 | 2.353 | 0.0374 |  | *ycf37* | 0.0364 | 2.4222 | 0.0882 |
| *rps9* | 0.0424 | 2.1706 | 0.0920 |  | *ycf39* | 0.035 | 2.145 | 0.0751 |
| *rps10* | 0.0271 | 2.6457 | 0.0717 |  | *ycf41* | 0.1187 | 1.3018 | 0.1545 |
| *rps11* | 0.0212 | 2.7053 | 0.0574 |  | *ycf42* | 0.0211 | 2.7221 | 0.0574 |
| *rps12* | 0.0056 | 2.0302 | 0.0114 |  | *ycf46* | 0.0242 | 2.9544 | 0.0715 |
| *rps13* | 0.0312 | 2.4283 | 0.0758 |  | *ycf47* | 0.0333 | 2.1976 | 0.0732 |
| *rps14* | 0.036 | 2.3475 | 0.0845 |  | *ycf65* | 0.0327 | 2.3935 | 0.0783 |
| *rps16* | 0.0323 | 2.5841 | 0.0835 |  | *ycf66* | 0.0299 | 1.6523 | 0.0494 |
| *rps17* | 0.0373 | 1.4862 | 0.0554 |  |  |  |  |  |

dN: nonsynonymous substitutions; dS: synonymous substitutions
