# Supplementary figures and images for: The organellar genomes of Silvetia siliquosa (Fucales, Phaeophyceae) and comparative analyses of the brown algae
Source: PLoS One. 2022 Jun 16;17(6):e0269631. doi: 10.1371/journal.pone.0269631 (PMC9202911; doi:10.1371/journal.pone.0269631)

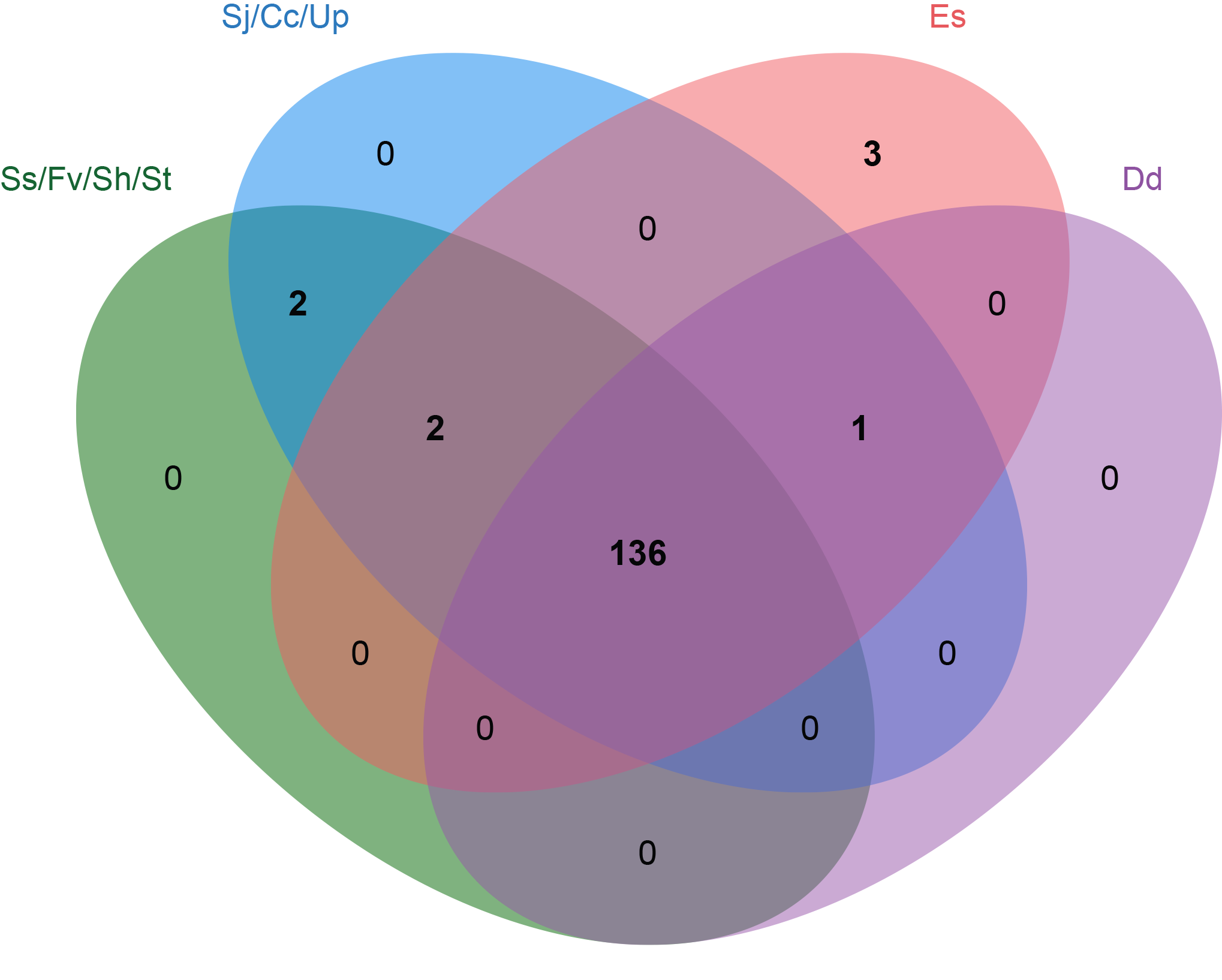

Supplement: S1 Fig — The numbers in the Venn diagram represent the number of shared and/or unique gene. (TIF) [file pone.0269631.s005.tif]

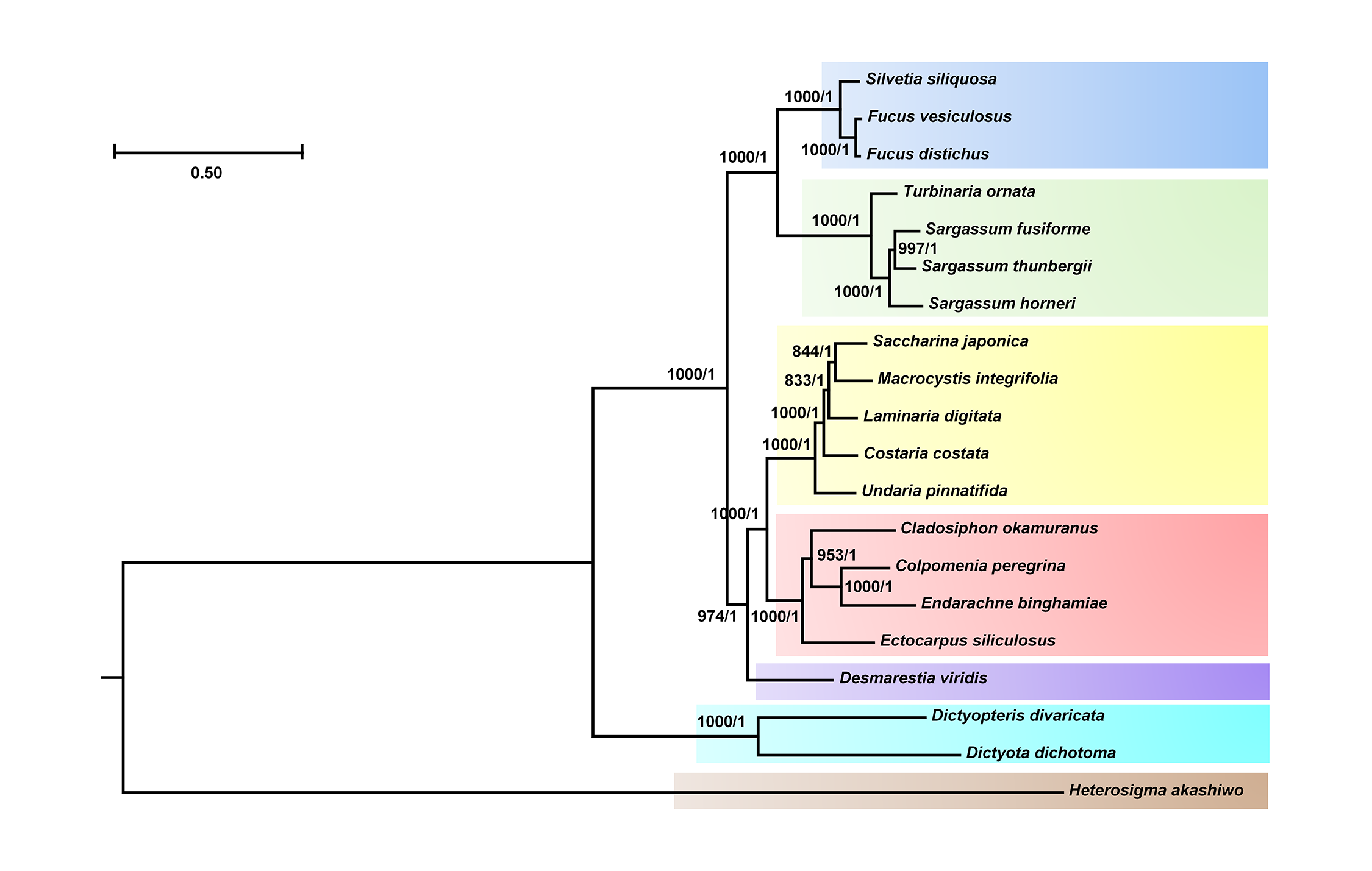

Supplement: S2 Fig — The numbers near each node are bootstrap support values in ML and posterior probability in BI with H. akashiwo as outgroup. (TIF) [file pone.0269631.s006.tif]

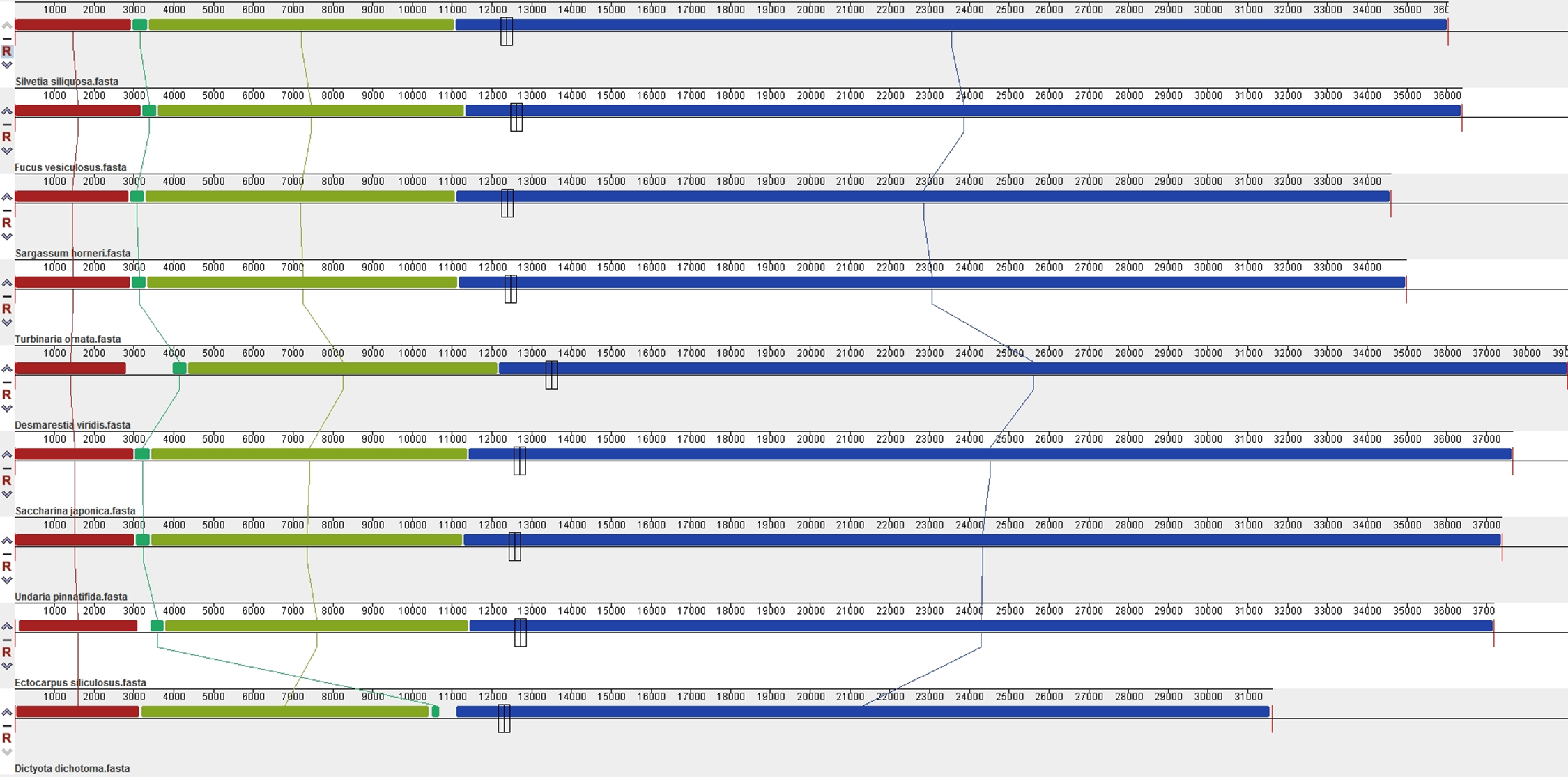

Supplement: S3 Fig — (TIF) [file pone.0269631.s007.tif]
