# Supplementary material for: The organellar genomes of Silvetia siliquosa (Fucales, Phaeophyceae) and comparative analyses of the brown algae
Source: PLoS One. 2022 Jun 16;17(6):e0269631. doi: 10.1371/journal.pone.0269631 (PMC9202911; doi:10.1371/journal.pone.0269631)
Supplement: Attachment — Submitted filename: Response to Reviewers.docx [file pone.0269631.s008.docx]

**To Reviewer #1:**

**Main comments/questions:**

**Question 1:** Line 115: Why was this particular set of genes chosen for molecular dating? Is it because they are slow-evolving and well conserved across diverse taxa, or are they simply the ones available for most taxa, as indicated later in Line 239? I believe it would be clearer if the reason behind the gene set selection was stated clearly in the Materials and Methods section.

**Response**: Thanks for this valuable comment. We selected 15 taxa with both mitochondrial and chloroplast genomes to be sequenced. These genes (*cox1*, *cox*3, *nad1*, *nad4*, *atp9*, *rbcL*, *psbA*, and *atpB*) are used to molecular dating due to their high conservation and slow evolutionary rate among brown algal taxa, and they are widely used in the estimation of divergence time in brown algae (Silberfeld et al., 2010; Starko et al., 2019). As suggested, we added instructions in the materials and methods as “These genes were highly conserved and slow-evolving”.

**Reference：**

Silberfeld T, Leigh JW, Verbruggen H, Cruaud C, Reviers BD, Rousseau F. A multi-locus time-calibrated phylogeny of the brown algae (Heterokonta, Ochrophyta, Phaeophyceae): Investigating the evolutionary nature of the “brown algal crown radiation”. Mol Phylogenet Evol. 2010; 56: 659−674. doi: 10.1016/j.ympev.2010.04.020

Starko S, Gomez MS, Darby H, Demes KW, Kawai H, Yotsukura N, et al. A comprehensive kelp phylogeny sheds light on the evolution of an ecosystem. Mol Phylogenet Evol. 2019; 136: 138−150. doi: 10.1016/j.ympev.2019.04.012

**Question 2:** Line 146: The taxonomic name Fucophycidae is used here and later in line 152, however, there is no explanation for what is included in this taxon anywhere in the text or figures. I would suggest that a sentence explaining what exactly are the Fucophycidae should be added at some point in the Introduction, or, alternatively, this taxon could simply be marked on Figure 3 along with order names.

**Response**: As suggested, we have marked the class names (Fucophycidae and Phaeophyceae) in Figure 3.

**Question 3:** Lines 149-155: This fragment suggests that there are three pairs of overlapping genes in S. siliquosa; however, in Table 1, S. siliquosa is listed as having 10 such pairs. Why are only three of them described in detail in text? Are they the only ones conserved across Fucophycidae, while all others are genus or species-specific?

**Response**: Yes, although 10 pairs of overlapping regions were detected in the *S. siliquosa* mitochondrial genome, these three pairs of overlapping regions were found to be highly conserved among Fucophycidae species by comparison with other brown algae.

**Question 4:** Lines 212-217: More a comment than question, but in case of the missing chloroplast genes, studies of genomic and transcriptomic data from other algal lineages (e.g. Pelagophyceae – Ong et al. 2010, doi:10.1111/j.1529-8817.2010.00841x, or Dictyochophyceae – Han et al. 2019, doi:10.1111/jpy.12904) have shown that chloroplast-to-nuclear genome transfer is rather frequent among ochrophytes, compared to total gene loss. Of course, a transcriptomic analysis would be far beyond the scope of this study (and not in good taste to request in a review), but I believe it would be worth mentioning that transfer to nucleus is the most plausible explanation for the variation in gene content in brown algal cp-genomes, especially considering that two of the genes missing in certain Phaeophyceae – specifically syfB and rpl32 – have been found to be transferred to the nucleus in some of the Dictyochophyceae (see Han et al. above).

**Response**: Thanks for this constructive comment. Horizontal gene transfer is usually determined based on homologous sequence alignment analysis. Currently, there are few available nuclear genome data for brown algae, so it is difficult to compare the homology between chloroplast and nuclear genomes. We also believe that with increasing genomic data, the veil of horizontal gene transfer events between organelles and nuclear genomes of brown algae will be uncovered.

**Minor (non-scientific) remarks:**

**Question 1：**Line 45: …accurate and deep insight (“deep” is twice in the manuscript)

**Response**: As suggested, we have deleted the extra word “deep”.

**Question 2：**Line 94: … progressive Mauve alignment algorithm (“the” after Mauve is redundant)

**Response**: As suggested, we have deleted the word “the”.

**Question 3：**Line 134: in “… in Tables 1 and 2 for this research”, I believe “analysis” would be a better-fitting word than “research”, which is more general

**Response**: Thanks, we changed the word “research” to “analysis”.

**Question 4：**Line 145: I believe “S. horneri” should be listed as “Sargassum horneri”, as this particular species has not been referred to before in the text – especially with the prior use of “S.” as Silvetia in the same sentence

**Response**: As suggested, we replaced ‘*S. horneri*” by using “*Sargassum horneri*.”

**Question 5：**Line 148: “…containing 94.31% of known brown algal genes and ORFs” – this phrase suggests that 94.31% of genes that are known from other brown algae is found in S. siliquosa – I believe you mean that 94.31% of its length are coding sequences – known brown algal genes and ORFs?

**Response**: Yes, we didn't make it clear. Accordingly, this sentence has been rephrased to “The mt-genome of *S. siliquosa* is gene dense, and the length of coding genes accounts for 94.31% of the total mt-genome, and non-coding regions accounts for only 5.69%, well within the range of Phaeophycean species (3.21-6.49%, Table 1).”

**Question 6：**Line 148: “…non-coding genes” – did you mean non-coding regions?

**Response**: Yes, as suggested, we have changed “non-coding genes” to “non-coding regions.”

**Question 7：**Lines 231-232: “The reconstructed phylogenetic tree …both formed one sister clade” – I believe it would be better to rephrase it as “…both together formed one clade”, or to make it even simpler - “…supported Laminariales and Ectocarpales as sister monophyletic groups.”

**Response**: As suggested, this sentence has been rephrased to “The reconstructed phylogenetic tree supported Laminariales and Ectocarpales as sister monophyletic groups.”

**Question 8：**Line 262: I would suggest using “brown algae” instead of Phaeophyta, as this name is not used anywhere else in the text, and even being a synonym of Phaeophyceae, it might just be distracting to a reader not proficient in taxonomy.

**Response**: Thanks, we changed “brown algae” to “Phaeophyceae species”.

**Question 9：**Line 287: “volvocine” should not be capitalized, as it is not a Latin name

**Response**: Thanks for this constructive comment, accordingly we changed “volvocine” to “*Volvulina compacta*”.

**Question 10：**Line 302: I believe the sentence ends after “…and S. thunbergii”, but the full stop is missing

**Response**: Thanks, we have modified it.

**Question 11：**Line 351: “…higher than that of *E. siliculosus*” – do you mean “higher than between any of these groups and *E. siliculosus*”?

**Response**: Yes, we have rephrased it accordingly.

**Question 12：**Line 357: “…supported in the Sphacelariales” – although it is not highly relevant to the work, I would suggest adding a hint at what the Sphacelariales are, as this name is not mentioned elsewhere in the text (e.g. “…supported in another phaeophycean order Sphacelariales”)

**Response**: Thanks for this comment. We have rephrased it accordingly.

**To Reviewer #2:**

**Specific comments:**

**Question 1：**line 43: I do not understand how information on organellar genomes are linked to conservation efforts. Besides offering greater access to variant positions, they offer little to no relevant functional performance information (in the brown seaweeds at least, because gene content is conserved).

**Response**: Thanks, although our study was not effective for the proliferation of this threatened algae, as a method of *ex situ* conservation, establishing gene bank of threatened algae can provide basic research data for the conservation of threatened algae.

**Question 2：**line 43: 53-55: see general comments on why this statement is not true. Ideally, the Rana et al. study should be retracted, as this claim was the basis and main conclusion for the paper.

**Response**: Thanks for this valuable comment, accordingly we deleted this citation and reference.

**Question 3：**line 55-58: We already know gene content is conserved, undermining this statement. As well, organellar genomes are fairly well characterized, with scope now capturing the breadth of Phaeophyceae since the starko et al. 2021 study. Evidently, however, organellar genomes remain to be sequenced in several less well studied orders.

**Response**: Thanks for this comment. We have rephrased it accordingly as follows:

“For example, designing molecular markers based on gene variable regions (nucleotide insertion/deletion) can be used for species identification [10, 17]. However, there is still limited genomic information in the brown alga, limiting our understanding of the taxonomic status and evolutionary history of the Phaeophyceae.”

**Reference:**

**10.** Yotsukura N, Shimizu T, Katayama T, Druehl LD. Mitochondrial DNA sequence variation of four *Saccharina* species (Laminariales, Phaeophyceae) growing in Japan. J Appl Phycol. 2010; 22: 243−251. doi: 10.1007/s10811-009-9452-7

**17.** Melton JT, Leliaert F, Tronholm A, Lopez-Bautista JM. The complete chloroplast and mitochondrial genomes of the green macroalga *Ulva* sp. UNA00071828 (Ulvophyceae, Chlorophyta). PLoS ONE. 2019; 10: e0121020. doi: 10.1371/journal.pone.0121020

**Question 4：**line 80: The authors here a priori assume organellar topology consistent with other species of Fucales. Looking at the genomes, clearly the authors correctly assembled the genomes. My recommendation for future work, however, is to employ different assembly methods to arrive at answers without any a priori assumptions made. For instance, the authors can elongate a seed sequence using NOVOPlasty, which confirms circularity, and should ensure to map reads back to the genome to ensure assembly errors are not present (i.e. coverage should be consistent, with no breaks in overlapping sequences).

**Response**: Thanks for this constructive comment.

**Question 5：**line 104: the authors should include other published genomes from other orders, see my general comment above. Desmarestia appears in Table S1, but why not in the phylogenetic tree

**Response**: Thanks, the phylogenetic tree containing Desmarestia can be found in Fig. S2.

**Question 6：**line 204-209: see comment above on genes that are incorrectly reported to be absent

**Response**: Thanks for your constructive comment. Starko et al. (2021) suggested that the loss of *ycf17* gene appear to be the result of annotation errors, but we used *ycf17* in *Saccharina japonica* (Genbank number: NC_013476) for homology comparison based on Nucleotide Blast, and this homologous gene was not found in the chloroplast genome of the reported order Fucales. In addition, how to find this gene in Fucales was not explained in Starko's article. None of the previously published chloroplast genomes of Fucales contain *ycf17* gene (Le Corguillé et al., 2009; Liu and Pang, 2016; Yang et al., 2016; Graf et al., 2017; Liu et al., 2018).

**Reference:**

Le Corguillé G, Pearson G, Valente M, Viegas C, Gschloessl B, Corre E, et al. Plastid genomes of two brown algae, *Ectocarpus siliculosus* and *Fucus vesiculosus*: further insights on the evolution of red-algal derived plastids. BMC Evol Biol. 2009; 9: 253. doi: 10.1186/1471-2148-9-253

Liu and Pang. Chloroplast genome of *Sargassum horneri* (Sargassaceae, Phaeophyceae): comparative chloroplast genomics of brown algae. J Appl Phycol. 2016; 28: 1419−1426. doi: 10.1007/s10811-015-0609-2

Yang JH, Graf L, Cho CH, Jeon BH, Kim JH, Yoon HS. Complete plastid genome of an ecologically important brown alga *Sargassum thunbergii* (Fucales, Phaeophyceae). Mar Genomics. 2016; 28: 17−20. doi: [10.1016/j.margen.2016.03.003](https://doi.org/10.1016/j.margen.2016.03.003)

Graf L, Kim YJ, Cho GY, Miller KA, Yoon HS. Plastid and mitochondrial genomes of *Coccophora langsdorfii* (Fucales, Phaeophyceae) and the utility of molecular markers. PLoS ONE. 2017; 12: e0187104. doi: 10.1371/journal.pone.0187104

Liu F, Pan J, Zhang ZS, Moejes FW. Organelle genomes of *Sargassum confusum* (Fucales, Phaeophyceae): mtDNA vs cpDNA. J Appl Phycol. 2018; 30: 2715−2722. doi: 10.1007/s10811-018-1461-y

**Question 7：**line 333-334: many more rearrangements depicted by Starko et al. 2021

**Response**: Sorry, we did not understand your question. Do you mean that the results here are wrong? However, in lines 333 to 334, we found rearrangement of four genes (atp8, rpl31, rps10, and atp9) in the mitochondrial genome of *D. dichotoma*, which is consistent with Starko's results (Starko et al., 2021; Fig. 4).

**Reference:**

Starko S, Bringloe TT, Gomez MS, Darby H, Graham SW, Martone PT. Genomic rearrangements and sequence evolution across brown algal organelles. Genome Biol Evol. 2021; 7: evab124. doi: 10.1093/gbe/evab124/6290714
