# Supplementary material for: The organellar genomes of Silvetia siliquosa (Fucales, Phaeophyceae) and comparative analyses of the brown algae
Source: PLoS One. 2022 Jun 16;17(6):e0269631. doi: 10.1371/journal.pone.0269631 (PMC9202911; doi:10.1371/journal.pone.0269631)
Supplement: Attachment — Submitted filename: Response to Reviewers.docx [file pone.0269631.s009.docx]

**To Reviewer #1:**

**Question 1:** Line 302 - "*S. thunbergia*" instead of "*thunbergii*"

**Response**: Thanks, we have modified it.

**To Reviewer #2:**

**General comments**

**Question 1:** The phylogenetic analyses are not taxonomically inclusive. What is the justification from the authors for not including other published genomes in their analyses, particularly from the orders Chordales (mito: MZ156045, MZ156050, MZ156063; plastid: MZ156027, MZ156037, MZ156030), Sphacelariales (mito: MZ156064; plastid: MZ156028), Ralfsiales (mito: MZ156065; plastid: NA); and Ishegeales (mito: MG940857; plastid: NA). I cannot think of a justifiable reason to not use all the available data for these inferences.

**Response**: The purpose of our phylogenetic tree is not to solve the evolutionary relationship between brown algae, but to solve the taxonomic status of *S. siliquosa* in brown algae, so we did not add additional taxa. Phylogenetic analysis revealed a close genetic relationship between *S. siliquosa* and *F. vesiculosus*, which diverged approximately 8 Mya (5.7–11.0 Mya), corresponding to the Late Miocene (5.3–11.6 Ma).

**Question 2:** The authors continue to reiterate gene loss information that I don’t believe is supported by their data. Unfortunately, as I cannot access the sequences for Silvetia, I cannot verify this. Taking the authors phylogenetic tree of mitochondrial markers, I assume Fucus is the closest relative with a published genome to verify their claims. Taking Fucus vesiculosus MG922855 then, one can take the ycf17 gene from another brown alga (I arbitrarily did this for Postelsia palmaeformis MZ156031) and map this sequence to MG922855 using built in geneious mapping algorithm or bbmap plugin. A gene sequence region is revealed positioned just before ycf19, with the start and stop positions slightly shifted compared to postelsia.
>ycf17_fucus_vesiculosus_ MG922855 (reversed)
ATGAATAAAGACTACAATTATAACAATTCTATAGAGTATAAAATAAGATGGGGGTTCTATTTAAAAAATGAAATTTTAAATGGTCGTGGTGCAATGATTTTATTAATAATAATAATATTATTAGAAATTTTTACACATAAAACTATAGTAAATTTAATCTTTCAAAGGTAA
You can then submit the translated sequence to interproscan (https://www.ebi.ac.uk/interpro/search/sequence/) and you will see both sequences from Fucus and Postelsia, while not predicted to be a specific gene, has nearly identical domains, one for chlorophyll a-b binding. The authors can also blast the translated sequence and see it matches ycf17 in other brown algae (a nucleotide blast does not come up with matches). Or, the authors can take ycf17 annotations from other brown seaweeds and align it with the above sequence. They will see the above sequence is indeed ycf17.
Similar exercises can be carried out in the other putatively lost genes, including ycf54 and petL for other orders such as Laminariales. The authors cite several studies publishing Fucales organellar genomes without ycf17; annotation algorithms are not fool proof, and we are prone to perpetuating errors if left unchecked. The authors can verify the presence of ycf17 in other fucales; Coccophora has a similar above sequence, also positioned prior to ycf19. Unless the authors have a compelling rebuttal, which I am of course open to, I must insist they do not continue to perpetuate errors in the literature regarding these lost genes.

**Response**: Thanks for this valuable comment. As suggested, we re-annotated the ycf17 gene in the *S. siliquosa* chloroplast genome and re-uploaded it to NCBI （Genebank number: MW485980）. In addition, we re-analyzed the gene components of the chloroplast genomes of brown algae.

**Question 3:** The authors are very descriptive in the discussion without providing any context for the observations, e.g. 145-193. Are these observations typical? What, if anything, does this tell us about the biology of brown seaweeds. If there is no bigger picture, what then is the relevance of these results? In particular, the authors missed an opportunity to describe divergence within the context of earth’s history, both in terms of climate and biome, or how evolutionary histories in different lineages shaped substitution rates.

**Response**: Thanks, we analyzed the gene composition of the mitochondrial and chloroplast genomes of *S. siliquosa* and compared them with those of other brown algae. However, our results did not find significant novelty, such as gene loss and gain, so we cannot discuss it in a better perspective. Our goal is to fully analyze the organelle genomes of common brown algae including the species we study, and to explore the changes of organelle structure.

**Question 4:** Please also upload your short read data to the short read archive, otherwise the genome assembly, the foundation of this work, is not verifiable or replicable.
**Response**: The genome sequences information of our organelles has been uploaded to NCBI, and you can query them by Genebank number MW485976 and MW485980, respectively. For the chloroplast genome, we re-annotated ycf17 according to your suggestion.

**Specific comments:**

**Question 1:** 48: Recent whole genome work calls into question this assumption of using organellar genomes for phylogenetic inferences. Phylogenetic inferences are hampered by uniparentally inherited genomes (i.e. organelles) in the presence of hybridization and organellar capture, which appears to the case in some brown algae (Bringloe et al. 2021, Journal of Phycology, Whole genome sequencing reveals..). In this case of the kelp Alaria, each genomic compartment (i.e. mitochondrial, plastid, and nuclear) revealed completely different topologies and evolutionary histories. It might be the case that relationships in closely related species of brown algae are not accurately reflected in organellar genomes if hybridizations is common, something we cannot easily evaluate or detect without whole genome data.

**Response**: Thanks for this constructive comment. For brown algae, the genomes of only 7 species have been sequenced (https://www.ncbi.nlm.nih.gov/genome/?term=txid2870[Organism:exp]), so it is difficult to construct the tree of life of brown algae. We also admit that the current organelle genome sequencing may not reflect the evolutionary history and topological relationship between brown algae, but for now, the construction of evolutionary tree using organelle genome is the most effective methods to solve the evolutionary relationship. In addition, the sequencing of *S. siliquosa* genome is ongoing, hoping to better solve the evolutionary relationship of brown algae in the future. We have rephrased it accordingly as follows:

“Complete organelle genome data can provide important reference for the phylogenetic construction of brown algae.”

**Question 2:** 52: what is meant here by “gene variable regions” and how does this relate to insertions or deletions, which are (as far as I know) extremely rare within genes in brown algal organellar genomes (since this would cause the gene to be non functional by shifting the reading frame).

**Response**: Thanks, we have rephrased it accordingly as follows:

“For example, designing molecular markers based on polymorphism can be used for species identification.”

**Question 3:** 54: Can the authors be more specific here. There is good coverage in fucales and Laminariales, but relatively little in less well studied brown algal orders. There are a lot of published organellar genomes, but they are severely taxonomically biased.

**Response**: Thanks, we have rephrased it accordingly as follows:

“Additional organelle genomes from novel taxa will not only provides data support for analyzing the structural variation of organelle genomes, but also advance our understanding of the evolution and diversity of brown algae.”

**Question 4:** 59-60: what is meant by typical brown algae?

**Response**: Typical brown algae refers to the common economic brown algae.

**Question 5:** 173-179: Here and elsewhere in the discussion, the material is very descriptive. If presenting this information, can the authors clarify its relevance? Are these observations abnormal or species compared to other species, brown algae or otherwise? I bigger picture is needed.

**Response**: Here, we would like to show the usage of start and stop codons in the mitochondrial genome of *S. siliquosa*, and compare it with other brown algae, and would like to present a comparative result to the reader, as the title of our paper also mentions "comparative analyses of the brown algae”.

**Question 6:** 205-210: see above comment on why ycf17 is indeed present in Fucales, and petL and ycf54 are present in laminariales.

**Response**: Thanks for this valuable comment, accordingly we have re-annotated the chloroplast genome of *S. siliquosa*, and this part of the content has been modified.

**Question 7:** 332: speaking to my previous comment, which the authors asked for clarification on, I would dispute the claim that mitochondrial genome architecture is highly conserved. One rearrangement is cited in the discussion, however, many are depicted in Starko et al 2021. Again, the constrained taxonomic scope of this paper is misleading.

**Response**: Thanks for this constructive comment, as you said, fewer taxa were used in the analysis of mitochondrial genome architecture and only one rearrangement was found, but there was nothing wrong with our results. Furthermore, our final conclusion is to illustrate that the structural variation of the chloroplast genome of the brown algae is significantly larger than that of the mitochondrial genome, which is consistent with the results of Starko et al (2021). We have rephrased it accordingly.

**Question 8:** 357-359: Not clear how rearrangements play a role in species divergence, can the authors explain this a bit further. Otherwise this comes across as wild speculation. If there is a compelling hypothesis here, it would be helpful for the authors to be clear on what is occurring and how it might be validated in future work.

**Response**: Thanks, this hypothesis was proposed because, based on rearrangement data, we found that brown algal chloroplast genomes exhibited many rearrangement and inversion events at the different order level, with less structural variation within the same order.

**Question 9:** Fig.6: The long and short single copy regions are out of order for Ectocarpus, making this figure harder to interpret in terms of smaller rearrangements (the figure implies the short and large single copy regions are inverted in this species). The authors should rearrange the figure so the long single copy region appears first in Ectocarpus, consistent with the other species depicted here.
What is the basis for the taxa selected in this figure? Why are other published arrangements not included? E.g. Sphacelariales, Desmarestiales, ect.

**Response**: Figure 6 has been modified as suggested. In addition, the taxa were chosen for the sole purpose of supporting or validating our results, and we found that the current data set is sufficient to support the conclusion that the chloroplast genomes of brown algae are more structurally variable than the mitochondrial genomes.
