# Supplementary material for: The organellar genomes of Silvetia siliquosa (Fucales, Phaeophyceae) and comparative analyses of the brown algae
Source: PLoS One. 2022 Jun 16;17(6):e0269631. doi: 10.1371/journal.pone.0269631 (PMC9202911; doi:10.1371/journal.pone.0269631)
Supplement: Attachment — Submitted filename: Response to Reviewers.docx [file pone.0269631.s010.docx]

**To Reviewer #1:**

**Review Comment:** All comments and suggestions have been addressed. I have submitted my recommendation to accept this work for publication.

**Response**: Thank you for reviewing this article. Thank you very much.

**To Reviewer #2:**

**General comments:** In this paper, De Duan et al. present the organellar genomes of the fucoidal species Silvetia siliquosa, which is endemic to the Northwest Pacific. The authors extend their analysis to other members of brown seaweed, analysing gene content, genome architecture, sequence evolution, and time calibrated phylogenetic placement. In my initial review, I pointed out several errors in interpreting putatively lost genes, and argued that many of the findings were already presented in more taxonomically inclusive datasets. In the most recent submission, the errors regarding gene loss have been corrected, but I still have some outstanding issues with the paper.

First, I have remained confused through this review process regarding the taxonomic scope of the paper. The authors insist the paper is about comparing the organellar genomes of S. siliquosa to other brown algae, specifically to resolve its phylogenetic placement (which they used to justify the taxonomic scope of their time calibrated tree), but they also analyse the organellar genomes of most (but not all) representative brown seaweeds with respect to genomic architecture and sequence evolution, and spend most of the discussion elaborating at this broader taxonomic scale. Because the scope of the paper extends beyond S. siliquosa, I remain confused as to why the authors did not draw in all the available genomes for brown seaweeds into their analyses. I have a fundamental problem with an analysis of the phaeophyceae that ignores some of the available data, especially at the ordinal level where differences in patterns are most likely to emerge. While I do not agree with the conflation of these objectives and how the data was gathered and analysed at the broader scale of all brown seaweeds, the editor will ultimately have to decide if this is acceptable for publication. Perhaps a compromise is for the authors to acknowledge in the manuscript that they did not analyse all the available brown algal genomes at the ordinal level (I listed these in my last review). This, at least, would make this aspect of the manuscript transparent to the reader.

**Response**: Thanks for this valuable comment. We added the deficiencies in our research in the conclusion section of the manuscript as follows：

“However, our study did not integrate all brown algae orders, and additional organellar genomes at the ordinal level are needed for further study of their organellar genome evolution.”

**General comments:** I also took issue with the broader implications and interpretations of this work in my previous review. I think this is because the manuscript is very descriptive. I wonder how useful or relevant it is to present information such as total gene length, space content, or overlap sizes, especially when these numbers are so highly conserved across brown seaweeds. I’m just not clear on how or why these details are relevant. But the authors are entitled to present this, and if the editor is fine with a descriptive study of this nature, then I respect that decision.

**Response**: Thank you, we believe that the description of the organelle genome is an important part of the analysis of the first published organelle genome, and lays the foundation for the subsequent analysis of the structural variation of the organelle genome.

**General comments:**While I commend the authors on making corrections regarding gene losses, I must insist (again) that they upload their short read data to the short read archive, in addition to their submission of the fully assembled genomes to genbank (which they have already done). The assembled genomes are an endpoint in your analysis, not the data you generated, the latter of which must be available for re-analysis. The raw data (the short reads you used to map and assemble the organellar genomes) are fundamental to the repeatability of this work (at least, insofar as it relates to reproducing the organellar genomes of S. siliquosa). This must be done before I can endorse for publication.

**Response**: Thank，the raw reads of *Silvetia siliquosa* organelle genomes have been deposited in the NCBI Sequence Read Archive under the BioProject number PRJNA824893, Sequence Read Archive accession numbers of mitochondrial and chloroplast genomes are SAMN27488512 and SAMN27488513, respectively.
